# Natural killer cells: From surface receptors to the cure of high‐risk leukemia (Ceppellini Lecture)

**DOI:** 10.1111/tan.13509

**Published:** 2019-03-25

**Authors:** Michela Falco, Daniela Pende, Enrico Munari, Paola Vacca, Maria C. Mingari, Lorenzo Moretta

**Affiliations:** ^1^ Laboratorio di Immunologia Clinica e Sperimentale IRCCS Istituto G. Gaslini Genoa Italy; ^2^ Laboratorio di Immunologia IRCCS Ospedale Policlinico San Martino Genoa Italy; ^3^ Department of Pathology IRCCS Sacro Cuore Don Calabria Hospital Negrar Italy; ^4^ Department of Immunology IRCCS Ospedale Pediatrico Bambino Gesù Rome Italy; ^5^ Department of Experimental Medicine (DIMES) and CEBR Università di Genova Genoa Italy

**Keywords:** activating NK receptors, acute high‐risk leukemia, hematopoietic stem cell transplantation, HLA class I, inhibitory checkpoints, killer immunoglobulin‐like receptors, NK alloreactivity, NK cells

## Abstract

Natural killer (NK) cells are innate immune effector cells involved in the first line of defense against viral infections and malignancies. In the last three decades, the identification of HLA class I‐specific inhibitory killer immunoglobulin‐like receptors (KIR) and of the main activating receptors has strongly improved our understanding of the mechanisms regulating NK cell functions. The increased knowledge on how NK cells discriminate healthy cells from damaged cells has made it possible to transfer basic research notions to clinical applications. Of particular relevance is the strong NK‐mediated anti‐leukemia effect in haploidentical hematopoietic stem cell transplantation to cure high‐risk leukemia.

## 1 INTRODUCTION

Natural killer (NK) cells share with T and B lymphocytes a CD34^pos^ common lymphoid precursor originated from hemopoietic stem cells (HSCs) and primarily located in the bone marrow. Their development occurs through coordinated differentiation and maturation steps that result in the progressive commitment toward the NK cell lineage and the acquisition of functional competence.^1^, ^2^ Recently, NK cells have been assigned to the family of developmentally related innate lymphoid cells (ILCs), which includes four major groups differing from each other on the basis of transcription factors relevant for their development, and the set of cytokines produced.^3^, ^4^

**Figure 1:**
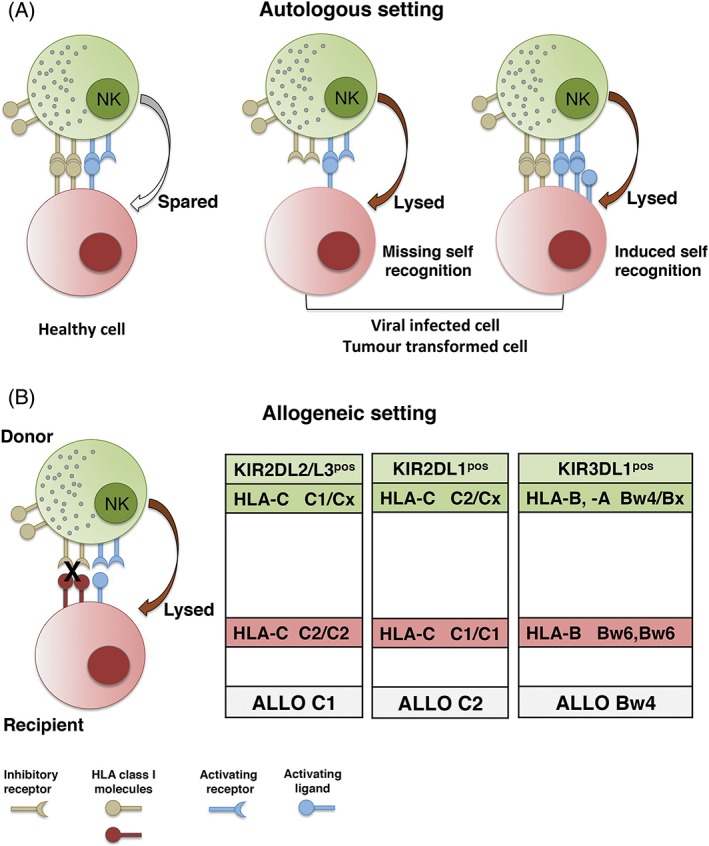
Natural killer (NK)‐mediated lysis in autologous and allogeneic settings. NK cells express inhibitory and activating receptors allowing to finely tune NK‐mediated cytotoxicity. A, In an autologous setting, NK cells spare healthy cells expressing HLA class I molecules whereas lyse autologous cells that, upon viral infection or tumor transformation, down‐regulate/loose HLA class I molecules or up‐regulate stress induced molecules. B, In an allogeneic setting, such as hematopoietic stem cell transplantation, donor NK cells expressing inhibitory receptor that recognize self‐HLA class I allotype not present on patient cells display alloreactivity

NK cells are important players of the innate immunity, able to sense and kill viral‐infected cells and neoplastic cells. The first NK cell functions described were the ability to lyse tumor target cell lines (eg, K562), even in the absence of previous stimulation, and to detect antibody‐coated target cells performing antibody dependent cell‐mediated cytotoxicity (ADCC) through the engagement of the low affinity receptor for Fc fragment of IgG (FcγRIIIa, CD16).^5^ Similar to CD8^pos^ cytotoxic T lymphocytes, NK cell cytolytic activity involves the polarized release of the content of lytic granules (including granzymes and perforin) at the immunological synapse at the NK‐target cell interface.^6^ A great input in the knowledge of NK cell function was reached in late 1980s. In mice, the “hybrid resistance phenomenon”, based on the observation that murine NK cells of F1 hybrid mice could reject the bone marrow derived from two inbred parental strains,^7^ suggested that NK cells are able to sense allelic major histocompatibility complex (MHC) polymorphisms. Concomitantly, in humans, the impact of MHC class I in the regulation of NK‐mediated recognition was suggested by experiments showing that interleukin‐2 (IL‐2) activated NK cells, expanded in mixed lymphocyte culture, could kill phytohemagglutinin (PHA) blasts obtained from the stimulating unrelated donor.^8^ The relevance of MHC molecules expression, as regulating elements of NK cytotoxicity, was also showed by the finding that murine NK cells could efficiently lyse a lymphoma cell line that had lost MHC class I expression while sparing the parental MHC class I‐positive cells. These results led to the formulation of the “missing self hypothesis”.^9^ Understanding of the molecular mechanisms governing NK‐mediated allorecognition was achieved by the identification of MHC class I specific inhibitory receptors in both mice and humans (Ly49 and KIR, respectively).^10^, ^11^, ^12^, ^13^, ^14^, ^15^, ^16^, ^17^ Since the discovery of the first inhibitory receptors, many molecules have been identified and characterized, leading to a better knowledge of the mechanisms governing NK cell functions. In particular, it was evident that the existence of an “off” signal preventing NK‐cell activation would imply the existence of an “on” signal generated on NK cell interaction with target cells. Many triggering NK receptors have been discovered including NKp46, NKp44, and NKp30 (collectively called natural cytotoxicity receptors, NCR), NKG2D, DNAM‐1, and 2B4.^18^, ^19^, ^20^ During the process of NK cell education, the interaction between HLA class I specific inhibitory NK receptors (iNKRs) and their cognate ligands sets the threshold of NK cell activation. Thus, a full maturation is achieved only by NK cells expressing at least one inhibitory receptor recognizing self‐HLA class I molecules (self‐iNKR) (Figure 1A). iNKR, specific for self‐HLA class I molecules expressed on healthy cells, prevent autoimmune reactions, especially in the context of inflammation in which NK cell function can be up‐regulated by the presence of cytokines and/or ligands of the activating receptors may be expressed in tissues. On the other hand, NK cells that do not express self‐iNKR remain anergic and do not react against autologous healthy cells.^21^, ^22^, ^23^ The capability of exerting cytolytic activity against virus infected or tumor cells is related to the ability of NK cells to sense, on target cells, the reduction/absence of HLA class I molecules (“missing self recognition”) and the upregulation of ligands for activating receptors (“induced self recognition”) (Figure 1A).^24^, ^25^ The most relevant iNKR are represented by the inhibitory KIRs (iKIRs), recognizing shared motifs of HLA‐A, ‐B, and ‐C allotypes (referred to as KIR‐ligands, KIR‐L), the heterodimer CD94:NKG2A that recognizes the non‐classical HLA‐E molecule, and the leukocyte immunoglobulin‐like receptor 1 that broadly reacts with several HLA class I allotypes.^17^, ^26^, ^27^

In peripheral blood, two different NK cell subsets can be identified on the basis of the surface density of CD56 expression. While CD56^dim^ are largely predominant, a minority (5%‐15%) is represented by CD56^bright^. CD56^dim^ NK cells are CD16^pos^ KIR^pos/neg^ CD94^pos^ (paired with either NKG2A or NKG2C), mediate cytotoxicity responses and release cytokines. CD56^bright^ NK cells are CD16^neg^ KIR^neg^ CD94/NKG2A^pos^, are non cytolytic, and produce high levels of proinflammatory cytokines.^28^, ^29^, ^30^ Several evidences indicate that CD56^bright^ give rise to CD56^dim^ NK cells.^31^ Another relevant difference between these two NK cell subsets is represented by the pattern of homing molecules expressed at their surface. Indeed, while CD56^high^ CD16^low^ express CCR7, required to reach secondary lymphoid organs, CD56^low^ CD16^high^ express CXCR1, CXCR2, and CXCR3 allowing their migration toward sites of inflammation. The CD56^bright^CD16^neg^ NK cells are more abundant in tissues.^32^

NK cells can produce a variety of cytokines and chemokines that regulate both innate and adaptive immune responses. Numerous studies showed that NK cells can interact with other cell types, in particular with dendritic cells (DCs).^33^ The NK/DC crosstalk can influence not only innate immune responses within inflamed tissues but also the subsequent adaptive immune response in secondary lymphoid organs.^34^ Importantly, interferon gamma (IFN‐γ) secreted by NK cells can lead to enhanced expression of HLA class I on target cells and/or HLA class II on antigen presenting cells, resulting in efficient stimulation of T cell–mediated immunity.^22^

## 2 NK CELL RECEPTORS RECOGNIZING HLA CLASS I MOLECULES

As mentioned previously, the ligand of the heterodimer CD94:NKG2A is HLA‐E, a non‐classical HLA class I molecule, expressed by virtually all cells.^35^ Because the most relevant peptides presented by HLA‐E derive from the classical HLA class I leader sequences,^27^ the level of HLA‐E expression mirrors the overall amount of HLA class I. Thus, NK cells, through CD94:NKG2A/HLA‐E interactions, monitor possible HLA class I decreases caused by infection or tumor transformation. Regarding iKIRs, the most relevant are KIR2DL1, KIR2DL2, and KIR2DL3 recognizing HLA‐C allotypes on the basis of the dimorphism at position 80, KIR3DL1 binding to HLA‐B and HLA‐A molecules sharing the Bw4 public epitope, and KIR3DL2 specific for HLA‐A*03 and ‐A*11 allotypes and HLA‐F.^36^ In particular, KIR2DL2 and KIR2DL3 recognize HLA‐C allotypes carrying asparagine 80 (HLA‐C C1 epitope) while KIR2DL1 bind HLA‐C allotypes sharing lysine 80 (HLA‐C C2 epitope).^12^, ^17^, ^37^, ^38^, ^39^, ^40^, ^41^ The extracellular regions of iKIR include 2 or 3 Ig‐like domains and are involved in ligand recognition. Their long cytoplasmic tails are characterized by immunoreceptor tyrosine‐based inhibitory motifs (ITIMs) that, upon KIR/KIR‐L recognition, recruit tyrosine phosphatases responsible of the inhibitory signals, switching off NK cell responses.^42^

Activating KIRs (aKIRs) and CD94:NKG2C represent the activating counterparts of HLA class I specific inhibitory receptors.^43^, ^44^, ^45^ The aKIRs display a short cytoplasmic tail, lacking ITIM, and carry a positively charged amino acidic residue in the transmembrane region that allows the interaction with KARAP/DAP‐12, adaptor molecules relevant for the activating signaling.^46^, ^47^ Despite structural similarities with their inhibitory counterparts, the specificity of aKIRs remained elusive for many years. Only during the last decade, the ligands for KIR2DS1, KIR2DS2, KIR2DS4, KIR2DS5, and KIR3DS1 have been identified.^48^, ^49^, ^50^, ^51^, ^52^, ^53^

### 2.1 *KIR* genes

The *KIR* gene family maps on chromosome 19 (19q14.3) and includes 13 functional *KIR* genes and 2 pseudogenes. *KIR* genes display an extremely high level of polymorphism, which is second only to that of *HLA* genes.^54^, ^55^, ^56^, ^57^, ^58^, ^59^ Notably, *KIR* variability is achieved by haplotype diversity (including variation in both gene content and gene copy number) and by allelic polymorphism. Nevertheless, the presence of four genes, collectively called framework genes, represents a conserved feature of *KIR* haplotypes. In particular, *KIR3DL3* ‐ *KIR3DP1* and *KIR2DL4* ‐ *KIR3DL2* pairs mark the ends of centromeric and telomeric regions, respectively.^60^ Based on the *KIR* gene content, two groups of haplotypes, namely “A” and “B”, have been identified. *KIR* A haplotypes are characterized by a fixed gene content including *KIR3DL3*, *KIR2DL3*, *KIR2DP1*, *KIR2DL1*, *KIR3DP1*, *KIR2DL4*, *KIR3DL1*, *KIR2DS4*, and *KIR3DL2.* Thus, A haplotypes are mainly encoding iKIRs able to sense all KIR ligands. On the contrary, *KIR* B haplotypes display a high gene content variation and include at least one of the following *KIR*: *KIR2DS2*, *KIR2DL2*, *KIR2DL5B*, *KIR2DS3*, *KIR3DS1*, *KIR2DL5A*, *KIR2DS5*, and *KIR2DS1.* 58, 60, 61 Notably, *KIR* A and B haplotypes have been detected in all human populations, although with different frequencies.^58^, ^62^, ^63^ *KIR* alleles may differ in exons coding for extracellular, transmembrane, or cytoplasmic regions. Notably, several polymorphisms at each of these three regions have been associated with significant biological consequences. Indeed, amino acidic variations determining intracellular retention or low expression of KIR, variability in ligand affinity, and diversity in signal transduction capability have been described.^64^, ^65^, ^66^, ^67^, ^68^

## 3 REGULATION OF NK CELL FUNCTION: ROLE OF INHIBITORY AND ACTIVATING RECEPTORS

Among different donors, a high degree of variability of NK cell phenotypes can be detected. During NK cell development, the diversity of NK cell receptor repertoire is primarily determined by *KIR/* *HLA* class I gene variability and by the clonal expression of KIR and NKG2 receptors, that are epigenetically regulated by DNA methylation and by noncoding RNAs.^69^, ^70^ Following the rules of NK cell “education”, dictating that each NK cell should express at least one inhibitory receptor for self‐HLA to become fully functional, highly stochastic, but self‐tolerant NK cell repertoires are generated.^36^, ^71^, ^72^, ^73^ The various KIRs are expressed on NK cell fractions. At single cell level, different numbers of iKIR can be detected, including also cells expressing only one iKIR specific for self‐HLA (self‐iKIR), in the absence of CD94/NKG2A.^74^ These NK cells can sense downregulation/loss of even a single HLA allotype, a common strategy exploited by viruses or cancer to escape immune surveillance.^75^, ^76^, ^77^ In the process of NK cell education, opposite to iKIR, aKIR induce down‐regulation of NK cell responsiveness if engaged by their cognate ligand. An example is represented by KIR2DS1. KIR2DS1^pos^ NK cells are educated in HLA‐C C1/Cx donors while they are anergic in HLA‐C C2/C2 individuals.^78^ Moreover, relevant changes in the NK repertoire can be induced by environmental factors, and in particular by human cytomegalovirus (HCMV) infection. Indeed, HCMV is a potent driver of NK cell maturation imprinting an “adaptive” or “memory‐like” phenotype, characterized by expansion of CD56^dim^ iKIR^pos^ (predominantly self HLA‐C specific KIR2DL) NKG2C^bright^ NKG2A^neg^ CD57^pos^ cell subset. Notably, CD57 expression marks terminally differentiated stages.^29^, ^79^, ^80^, ^81^

### 3.1 Non HLA‐specific activating receptors

In addition to the HLA‐specific receptors, NK cells express another important set of receptors and co‐receptors, which play a central role in NK cell activation on engagement with specific ligands on target cells. The major NK activating receptors involved in cancer cell recognition and killing induction are represented by NCR (comprising NKp46, NKp44, and NKp30), NKG2D (CD314), and DNAM‐1 (CD226).^18^, ^19^, ^82^, ^83^, ^84^, ^85^, ^86^ While NKp46 and NKp30 are present on resting NK cells and maintained upon activation, NKp44 expression is confined to activated NK cells. Originally discovered as NK‐specific triggering receptors, recently NCR expression has been detected also on some ILCs.^3^, ^4^, ^87^ In general, the ligands of these activating receptors are either upregulated or expressed de novo on “stressed” cells. Regarding the NCR ligands, B7‐H6 and BAT3 are recognized by NKp30, while MLL5 by NKp44. Notably, the membrane‐associated ligand of NKp46, a receptor which plays a major role in killing different tumor cells including leukemia blasts, is still undefined. It should also be mentioned that soluble NCR ligands have recently been discovered. They include the complement factor P for NKp46, the platelet‐derived growth factor P and Nidogen‐1 for NKp44.^88^, ^89^, ^90^ Moreover, NKG2D specifically binds to MICA/B and ULBPs, and DNAM‐1 is specific for poliovirus receptor and nectin‐2, both components of the cellular junctions.^91^, ^92^, ^93^ The function of these main activating receptors can be amplified by the signaling lymphocyte activation molecule family receptors, whose expression is confined to hematological cells. Among these co‐receptors, 2B4 recognizes CD48, and NTB‐A mediates homophilic interactions.^94^, ^95^, ^96^ A potent agonistic role is also played by TLR/TLR‐L interactions.^97^

Notably, the interactions of both inhibitory and activating receptors with their ligands represent important checkpoints finely tuning NK‐cell activation and function.^98^ While inhibitory interactions predominate when NK cells encounter autologous healthy cells, damaged cells can be susceptible to NK cell lysis through the mechanisms of “missing self recognition” and/or “induced self recognition” (Figure 1A). In a non‐autologous setting (such as allogeneic hematopoietic stem cell transplantation, HSCT) “missing self recognition” can occur when donor is characterized by an NK cell subset (referred to as alloreactive NK subset) expressing exclusively “educated” iKIR(s) which do not recognize any of the HLA class I molecules present in allogeneic cells (Figure 1B). The alloreactive NK cell subset can be identified and its size measured by multi‐color flow‐cytometry. This approach is based on staining the iKIR specific for the mismatched KIR‐L with a mAb conjugated with a given fluorochrome while the iKIR recognizing the allogeneic KIR‐L and CD94/NKG2A with a different fluorochrome. Of note, the positive contribution of aKIR can also be evaluated.^78^, ^93^, ^99^

## 4 EXPRESSION OF INHIBITORY CHECKPOINTS IN NK CELLS AND THEIR LIGANDS IN TUMORS

As illustrated previously, NK cell activation is controlled by HLA class I‐specific inhibitory receptors. With few exceptions (small subsets of T cells and ILC) they are confined to NK cells, so that their regulatory effect is exerted primarily on these cells. However, additional inhibitory checkpoints that play an important role in maintaining the immune response homeostasis have recently been detected in NK cells. These include PD‐1, TIGIT, TIM‐3 and so forth (see below). Notably, these inhibitory checkpoints are not constitutively expressed by resting NK cells, but they may be induced in pathological conditions, primarily in tumors and CMV infections and, on interaction with their specific ligands, they can inhibit NK cell function.

PD‐1 is a major checkpoint primarily expressed by activated NK cells which controls excessive T cell activation. PD‐1 is physiologically involved in maintaining the homeostasis of immune responses and in inducing peripheral T cell tolerance. Recent studies in ovarian carcinoma patients showed the presence of PD‐1^pos^ NK cells (confined to mature CD56^dim^ cells). These cells, isolated from patients, did not kill PD‐L1^pos^ tumor cells, while cytotoxicity could be restored in the presence of mAbs disrupting the PD‐1/PD‐L1 interaction.^100^ PD‐1^pos^ NK cells were also found in patients with Kaposi sarcoma and Hodgkin lymphoma.^101^, ^102^ Although, the molecular mechanism(s) involved in PD‐1 expression at the NK cell surface have not been identified yet, a pool of PD‐1 protein and mRNA has been identified in resting NK cells, suggesting the possibility of a prompt surface expression following appropriate stimulation.^103^ Other inhibitory checkpoints are expressed by NK cells (in addition to T‐lymphocytes) such as TIGIT and CD96, belonging to the same Ig superfamily of the activating receptor DNAM‐1.

TIGIT may be expressed by NK cells associated to colon‐rectal tumors. A recent report has suggested that its targeting with specific mAbs may unleash T and NK cell anti‐tumor activity and prevent NK cell exhaustion.^104^ Other inhibitory checkpoints that may be expressed by NK cells are LAG‐3 and TIM‐3. While the inhibitory activity of LAG‐3 on T cell activation has been showed, the effect on NK cell function is still undefined. TIM‐3 may be co‐expressed with PD‐1. In preclinical models, blockade of TIM‐3 restores T cell function and induces increases in NK cell cytotoxicity.^105^ A particularly important inhibitory checkpoint, the IL1R8, belongs to the IL1 receptor family and is a component of the human IL37 receptor. It appears to play a central physiologic role in dampening excessive inflammatory responses.^106^ As shown by a recent study, IL1R8 is highly expressed in NK cells and exerts a potent inhibitory control on NK cell activation, proliferation, and function.^107^ Remarkably, in a murine model, NK cells lacking IL1R8 prevented the growth of carcinogen‐induced hepatocarcinoma, showing that its blockade could unleash NK cells and promote strong anti‐tumor activity.

A large body of evidences indicates that immunotherapy with specific mAbs disrupting the PD‐1/PD‐L1 axis is highly effective in different tumors and represents a true revolution in tumor therapy. The spectrum of advanced malignancies in which the use of mAbs targeting PD‐1 or PD‐L1 is indicated has broadened, including non‐small lung cancer, melanoma, urothelial tumors, renal cell carcinoma, Hodgkin lymphoma, head and neck cancer, gastric tumor and so forth.^108^ Despite the unprecedented success in tumor therapy, a large number of tumor patients still do not benefit from this class of agents. In this context, the possibility to predict tumor response to therapy with PD‐1/PD‐L1 blocking agents represents a major research focus. At the present, the best recognized parameter of response is PD‐L1 expression in tumors, representing an approved guide for treatment decision. While the clinical usefulness of PD‐L1 expression as a suitable biomarker for response to treatment is clear, its predictive value is still unsatisfactory. Relevant limitations include intra‐ and inter‐tumor heterogeneity and technical issues related to the use of different mAbs and diagnostic material (cytology, diagnostic biopsies vs surgical specimens).^109^, ^110^ Thus, research in progress is focused on finding additional checkpoints to be exploited for targeting with therapeutic antibodies used alone or in combination. In this context, a promising progress to this aim has recently been reported by Andrè et al, showing that mAbs to NKG2A, used alone or in combination with other checkpoint inhibitors or with mAbs specific for tumor antigens, can lead to important results in murine models as well as in preliminary clinical studies.^111^, ^112^ In this context, it is noteworthy that NKG2A, while constitutively expressed by a large fraction of NK cells, can be induced also in T cells on prolonged antigenic stimulation or exposure to TGF‐β.^113^, ^114^ De novo expression of NKG2A leads to the impairment of T cell function. Accordingly, blocking of NKG2A may unleash not only NK, but also T lymphocytes with potential anti‐tumor activity. In this context, most human highly aggressive cancers express HLA‐E (the NKG2A ligand) and could also express PD‐L1. Accordingly, in vitro experiments indicated that IFN‐γ production and cytolytic activity of NK and T cells against HLA‐E^pos^ tumor cells were restored upon masking NKG2A. Moreover, in case of co‐expression of PD‐1 optimal functional recovery was documented in combination with mAbs disrupting the PD‐1/PD‐L1 axis. Another interesting and promising approach is based on the combined use of anti‐NKG2A and mAbs directed to tumor cell surface antigens. The efficacy of these mAbs is primarily dependent on NK‐mediated ADCC via CD16 receptor. Thus, unlocking the NK cytotoxicity with anti‐NKG2A mAbs allows NK cells to kill mAb‐opsonized tumor cells via their CD16. Importantly, in preliminary clinical studies (a phase II trial) the use of the anti‐NKG2A‐monalizumab in combination with an anti‐epidermal growth factor receptor (cetuximab) provided encouraging results in patients with head and neck tumors. These studies of combination therapies underscore the importance of harnessing NK cells in tumor therapies, while the various immunotherapeutic strategies have been so far aimed at potentiating T cell responses.

## 5 RELEVANCE OF NK CELLS IN TRANSPLANTATIONS

For patients with acute high‐risk leukemias, HSCT is the life‐saving therapy. HLA‐identical sibling or HLA 10/10 allelic matched unrelated donors represent the first donor choice. However, only 2 out of 3 patients find an HLA compatible donor, and this proportion can be even lower for patients of certain ethnic groups. If an HLA‐matched donor is unavailable, or patient is in urgent need of HSCT (eg, high risk of relapse), a haploidentical donor (a relative sharing an HLA haplotype with the patient) is a suitable alternative. Haploidentical‐HSCT (haplo‐HSCT) became successful in 1990s by intensifying the conditioning regimen to prevent graft rejection, using extensive T‐cell depletion to avoid graft vs host disease (GVHD), and a large donor graft inoculum of CD34^pos^ cells (“mega‐doses”).^115^, ^116^ This graft mainly relies on NK cells, representing the first lymphocyte population reconstituting the patients.^99^, ^117^, ^118^ A more favorable clinical outcome has been associated with donor NK alloreactivity (presence of KIR/KIR‐L mismatch in GvH direction).^117^, ^119^, ^120^, ^121^, ^122^, ^123^, ^124^, ^125^, ^126^ Indeed, donor‐derived alloreactive NK cells could play a crucial role in the eradication of leukemia blasts (graft vs leukemia, GvL, effect). Moreover, alloreactive NK subsets can also eliminate residual recipient DCs and T lymphocytes, thus preventing GvHD and graft rejection, respectively.^117^, ^127^ Notably, in this transplantation setting, the KIR repertoire of NK cells reconstituted from the donor CD34^pos^ precursors was mirroring that of the donor, suggesting that NK cell education occurred primarily via HLA expressed on donor derived cells, possibly due to the “mega‐doses” of donor cells infused. In CD34^pos^ haplo‐HSCT, the emergence of fully functional, KIR^pos^ alloreactive NK cells may take 6 to 8 weeks, thus delaying their benefit in the GvL effect.^99^, ^126^ Moreover, although primary engraftment and low GvHD rate were achieved, the extensive T‐cell depletion caused a slow post‐transplant immune recovery leading to viral reactivation and opportunistic infections. Since 2010, the use of a novel graft manipulation approach, based on the selective depletion of α/β T cells and B cells, allowed the infusion of mature donor NK cells and γ/δ T cells together with HSC precursors.^125^, ^128^, ^129^, ^130^ In this setting, NK cells can promptly exert an immediate anti‐leukemia effect soon after transplantation, before the emergence of NK cells differentiating from donor HSC. Alternatively, un‐manipulated T replete grafts can be performed, with high dose post‐transplant cyclophosphamide (PT‐Cy) to eliminate rapidly dividing donor T cells recognizing the HLA mismatched recipient and thus controlling GvHD.^131^, ^132^ It has recently been showed that administration of PT‐Cy profoundly depletes the infused NK cells, strongly impairing alloreactive NK cell subset functions.^133^ Regarding donor selection, several studies have shown that patients transplanted from donors carrying the B/x genotype and a B content value ≥2, have a better clinical outcome emphasizing the relevant role of aKIRs.^124^, ^134^, ^135^, ^136^, ^137^

## 6 CONCLUSIONS

Cells of the innate immunity have been underestimated for many years. However, during the past decades, their crucial role in controlling most of infections was underscored. Regarding NK cells, they play a relevant role in defenses against viruses and in the control of tumor growth and metastasis. This NK cell function reflects not only their direct intervention against pathogens/tumors thanks to their cytolytic activity and release of pro‐inflammatory cytokines, but also their ability to shape efficient (Th1) adaptive responses. Along this line, therapeutic approaches aimed at harnessing NK cells or reconstituting their function compromised by the tumor microenvironment or by a hypoxic milieu, or inhibitory cytokines, and so forth may result useful, particularly in tumor therapy. Thus, mAbs masking inhibitory receptors including those inducible (primarily PD‐1), and those constitutively expressed by NK cells (KIR and NKG2A), offered an unprecedented tool for cancer treatment. In this context, the demonstration by our group that such inhibitory receptors may be expressed also by CD8^pos^ CTL, upon antigen or cytokine‐induced cell proliferation or exposure to TGF‐β, offered a clue for the important studies by Andrè et al of NKG2A blocking in tumor therapy. In addition, the recent data that PD‐1 may be expressed also by NK cells offered new perspectives in the treatment with anti‐PD1 of tumors lacking HLA class I, thus resulting undetectable by T lymphocytes. The haploidentical HSCT to cure high‐risk leukemia showed another great potential of NK cells, also underscoring the central role of KIR/HLA class I mismatches.

Another major expectation for the therapy of both hematologic malignancies and solid tumor is based on the use of NK cells expressing chimeric antigen receptors (CARs) specific for tumor antigens. Notably, CAR‐NK may well complement CAR‐T lymphocytes. Some advantages may even be envisaged such as the possibility of using third‐part, CAR‐NK cells, since, different from T cells, they do not cause GvHD. In addition, in view of their potent cytolytic activity (present also in case of down‐regulation of the tumor antigen targeted by CAR) and their particular homing capability, they might mediate a better anti‐tumor effect.

While we must look at the future and at the continuous advances in medical research, we should also look back and be aware that without the seminal contributions of a giant of the HLA field, Ruggero Ceppellini, our knowledge in immunology and transplantation would not have been so advanced. A special tribute also to another outstanding scientist, Alessandro Moretta, who recently passed away. His groundbreaking discovery of the major activating and inhibitory receptors described in the present review revolutionized the NK cell field and offered fundamental tools for the cure of otherwise incurable leukemia.

## CONFLICT OF INTEREST

The authors have declared no conflicting interests.
